# Effect of Prior Laser-Assisted In Situ Keratomileusis on the Calibration Accuracy of Extended Depth of Focus Intraocular Lenses: A Direct Comparative Study

**DOI:** 10.3390/jpm15070301

**Published:** 2025-07-10

**Authors:** I-Hung Lin, Chen-Cheng Chao, Chao-Kai Chang

**Affiliations:** 1 Nobel Eye Institute, Taipei 100008, Taiwan; petercard@gmail.com (I.-H.L.); aachengachen@gmail.com (C.-C.C.); 2 Graduate Institute of Clinical Medicine, College of Medicine, National Taiwan University, Taipei 100225, Taiwan; 3 Department of Ophthalmology, Taipei Medical University Hospital, Taipei 110301, Taiwan; 4 Department of Optometry, MacKay Junior College of Medicine, Nursing and Management, Taipei 112021, Taiwan; 5 Department of Optometry, Da-Yeh University, Chunghua 515006, Taiwan

**Keywords:** laser in situ keratomileusis, cataract, extended depth of focus, absolute prediction error

## Abstract

**Background:** Personalized precision medicine has become a prevailing trend and applies to the selection of intraocular lenses (IOLs) for cataract surgery based on the unique corneal morphology of each person. The choice of presbyopia-correcting IOLs for post-laser-assisted in situ keratomileusis (LASIK) cataract surgery is a significant concern. However, few direct comparison studies exist between eyes with and without LASIK history. We analyzed the performance of extended depth of focus (EDOF) IOL implantation in these two groups. **Methods:** In this retrospective single-center study, we included patients with or without previous LASIK who underwent cataract surgery and EDOF Symfony IOL implantation, with ≥1 follow up. All patients underwent optical biometry using the IOLMaster. IOL power was calculated using the Sanders Retzslaff Kraff/theoretical and Haigis-L formulas for patients without and with LASIK, respectively. Uncorrected distance visual acuity (UDVA), uncorrected near visual acuity (UNVA), refraction, and corneal tomography were recorded. The prediction error was the absolute difference between the postoperative sphere and target refraction. The right eyes of patients who met the inclusion criteria were selected for analysis. **Results:** Among the 321 recruited eyes, 18 underwent previous LASIK. After 1:3 age/sex matching, 17 LASIK and 49 non-LASIK eyes from 66 patients were analyzed. No significant preoperative differences existed in target refraction, spherical equivalent, or best-corrected visual acuity. All surgical procedures were uneventful. LASIK exhibited non-inferiority to non-LASIK for predictive refraction error and UNVA. An age/sex-matched regression analysis indicated no UDVA superiority between the two groups. **Conclusions:** Previous LASIK may have no discernible effect on the visual performance of presbyopia-correcting EDOF IOLs with respect to the absolute refractive error, UNVA, and UDVA. Longer follow-up and larger-scale studies are required to further validate these results.

## 1. Introduction

Laser-assisted in situ keratomileusis (LASIK) is a widely utilized refractive surgery for correcting myopia and astigmatism by reshaping the corneal stroma with an excimer laser. This reshaping allows the focal point of incoming light to be realigned onto the retina, thereby improving visual acuity [1]. While LASIK is highly effective in reducing the dependence on corrective lenses, it can also alter corneal biomechanics and induce higher-order aberrations, such as coma and spherical aberration. These optical changes may influence visual quality, although their impact on contrast sensitivity remains debated [2].

With the aging of LASIK-treated individuals, an increasing number of these patients are now undergoing cataract surgery. Nowadays, personalized precision medicine has become a prevailing trend. The same applies to the selection of intraocular lenses (IOLs) for cataract surgery. We should choose the most suitable IOL based on the unique corneal morphology of each individual. The selection of an appropriate intraocular lens (IOL) for post-LASIK eyes presents unique challenges due to the altered corneal biomechanics and shape, which affects the accuracy of IOL power calculations. Traditional IOL calculation formulas often yield suboptimal refractive outcomes in post-LASIK eyes, necessitating specialized formulas or newer technologies to enhance predictability. Among various IOL options, multifocal and extended depth of focus (EDOF) IOLs have been proposed as potential solutions for presbyopia correction in these patients. However, concerns remain regarding their performance in eyes with previous LASIK, particularly in terms of visual quality, dysphotopsia, and contrast sensitivity.

Although some studies have suggested that multifocal IOLs can be successfully implanted in post-LASIK eyes under specific conditions—such as the presence of regular corneal astigmatism and the use of optimized IOL power calculation methods [3,4]—comparative data between post-LASIK and non-LASIK eyes remain limited. In particular, direct assessments of EDOF IOL performance in post-LASIK patients are scarce. Given the growing number of patients seeking spectacle independence after cataract surgery, a clearer understanding of how EDOF IOLs function in post-LASIK eyes is essential for optimizing surgical outcomes and patient satisfaction.

Against this background, this study aims to provide a comparative analysis of EDOF IOL implantation outcomes in patients with and without a history of LASIK. By evaluating the postoperative visual performance, we seek to determine whether EDOF IOLs are a viable option for individuals who have previously undergone LASIK.

## 2. Materials and Methods

### 2.1. Study Design and Ethical Approval

Patients who visited the Taipei Nobel Eye Clinic from 2022 to 2023 were consecutively enrolled. This study was approved by the Ethics Committee of the National Changhua University of Education (Changhua, Taiwan) and registered on ClinicalTrials.gov (identifier NCT06165796).

### 2.2. Patients

The inclusion criteria comprised the following: (1) the presence of cataracts in both eyes, (2) age between 50 and 80 years, and (3) a corrected distance visual acuity (CDVA) under 20/40 in both eyes. Phacoemulsification cataract surgery was performed in all patients with or without previous myopic LASIK surgery. The exclusion criteria included the following: (1) complicated cataracts, (2) corneal opacities or irregularities, (3) corneal astigmatism > 1.50 diopters, (4) dry eye (Schirmer’s test I ≤ 5 mm), (5) amblyopia, (6) anisometropia, (7) surgical complications such as posterior capsular bag rupture or vitreous loss, (8) IOL tilt or decentration, (9) coexisting ocular pathologies, (10) glaucoma, (11) non-dilating pupil, (12) history of intraocular surgery or retinopathy, (14) optic nerve or macular diseases, and (15) refusal or inability to maintain follow-up. Patients with optic zone decentration or irregular astigmatism in the LASIK group were excluded. Among patients who visited the Taipei Nobel Eye Clinic between 2022 and 2023, 331 eyes of 186 patients were recruited for this study.

### 2.3. Surgery

Clear corneal phacoemulsification and IOL implantation were performed by the same surgeon (Chao-Kai Chang). The surgical procedure involved the application of topical anesthesia, a three-step clear corneal incision (2.25 mm) at 180° (temporal in both eyes), a 5.0 mm continuous curvilinear capsulorhexis, phacoemulsification using the stop-and-chop technique, IOL implantation with an injector, IOL centration, and sutureless incisions. We exclusively used EDOF Symfony IOLs (Johnson & Johnson, Santa Ana, CA, USA).

### 2.4. Ophthalmic Examinations

The patients underwent comprehensive preoperative examinations and were subsequently assessed at 1 d, 1 week, and 1 month postoperatively. Each ophthalmological examination included tests for uncorrected distance visual acuity (UDVA) and CDVA at far and near distances, manifest refraction, biomicroscopy, and applanation tonometry. Corneal curvature values were exported from a Pentacam machine (Pentacam HR; Oculus GmbH, Wetzlar, Germany) using a Scheimpflug keratometry system. Fundus examinations were performed preoperatively. Preoperatively, all patients underwent optical biometry using the IOLMaster (IOLMaster 500, Carl Zeiss Meditec AG, Jena, Germany). The IOL power was calculated using the Sanders Retzlaff Kraff/theoretical (SRK/T) formula for patients without LASIK and the Haigis-L formula for patients with LASIK. The postoperative refraction target was emmetropia. This means that based on the post-IOL implantation refraction degree predicted by the IOLMaster, we chose an IOL as close to 0 degrees as possible for the IOL implantation, and that degree was defined as target refraction. Postoperatively, the IOL concentration was additionally evaluated using retro-illumination.

### 2.5. Statistical Analysis

The main post-surgery measurements used to compare the performance between patients with and without LASIK included the following: (i) ophthalmic examinations assessing UDVA, CDVA, and uncorrected near visual acuity (UNVA) at 40 cm and (ii) prediction error, defined as the absolute difference between the postoperative manifest sphere and target refraction. By using a model of generalized estimating equations (GEE), the regression analysis was adjusted for the following covariates: age, sex, axial length, anterior chamber depth (ACD), corneal curvature (K), IOL power, and preoperative sphere.

Non-inferiority tests [5] were conducted to assess whether the LASIK group’s performance was not inferior to that of the non-LASIK group with respect to three post-cataract surgery outcomes: absolute predictive error, UDVA, and UNVA. The null hypothesis posits that the difference in the group mean is not less than the inferiority margin, in contrast to the alternative hypothesis that the difference in the group mean is less than the inferiority margin. To assess the non-inferiority of the LASIK group compared with the non-LASIK group, a 95% confidence interval of the difference in the means of target refraction between the groups was constructed [5].

If the upper limit of the interval does not exceed the predefined non-inferiority margin of 0.5 diopters (D) in the prediction error, non-inferiority is confirmed. Similarly, for UNVA and UDVA, the predefined non-inferiority margins are both a 0.1 logarithm of the minimum angle of resolution (logMAR) [6,7].

## 3. Results

Among the 321 recruited eyes, 18 had undergone previous LASIK, while the remaining 303 had not. After 1:3 matching for age and sex, the analysis included a final sample of 17 LASIK eyes and 49 non-LASIK eyes from 66 patients. All the 17 LASIK eyes had received LASIK surgery at ages 20 to 50. Table 1 presents the demographic characteristics of the patients and preoperative visual acuity data. No significant preoperative differences were observed in the target refraction, spherical equivalent, or best-corrected visual acuity. The LASIK eyes exhibited longer axial lengths (27.57 ± 1.43 mm) compared with the non-LASIK eyes (25.63 ± 1.80 mm), and the ACD was also deeper in LASIK eyes (3.57 ± 0.36 mm) than in non-LASIK eyes (3.34 ± 0.37 mm). A corneal topography revealed flatter K1 and K2 values (38.07 ± 2.18 and 38.82 ± 2.17, respectively) in the LASIK eyes in comparison with the non-LASIK eyes (43.13 ± 1.55 and 44.46 ± 1.81, respectively) (Table 1). All surgical procedures were performed smoothly, and all IOLs were positioned within the capsular bag.

Table 2 presents the results of the non-inferiority tests at one month post-operation. For the UNVA, the mean was 0.315 ± 0.305 logMAR in the LASIK group and 0.736 ± 1.307 logMAR in the non-LASIK group. The respective non-inferiority margins exceeded the upper bounds of their corresponding confidence intervals (non-inferiority margins: 0.1; 95% CI: −0.8143, −0.0269), confirming the non-inferiority of the LASIK group compared with the non-LASIK group. For the predictive refraction error, the mean was 0.656 ± 0.264 D in the LASIK group and 0.551 ± 0.287 D in the non-LASIK group. The respective non-inferiority margins exceeded the upper bounds of their corresponding confidence intervals (non-inferiority margins: 0.5; 95% CI: −0.0442, 0.2541), confirming the non-inferiority of the LASIK group compared with the non-LASIK group. In contrast, for the UDVA, the mean was 0.300 ± 0.362 logMAR in the LASIK group and 0.117 ± 0.156 logMAR in the non-LASIK group. The non-inferiority margin fell within the interval, with the mean of the LASIK group being greater than that of the non-LASIK group (non-inferiority margins: 0.1; 95% CI: 0.0033, 0.3585). However, further age- and sex-matched regression analyses did not suggest that either group was significantly superior in terms of the UDVA (*p* = 0.063) (Table 3). It only revealed that the axial length has a significant influence on the UDVA (*p* = 0.034) (Table 3).

## 4. Discussion

In this retrospective single-center study, we compared the performance of EDOF IOLs after cataract surgery between eyes with and without a history of prior LASIK for myopia correction. The non-inferiority tests showed that the LASIK group was not inferior to the non-LASIK group for both the absolute predictive refractive error and the UNVA. Specifically, the upper bounds of the 95% confidence intervals for the mean differences were below the pre-defined non-inferiority margins of 0.5 D for refractive error and 0.1 logMAR for the UNVA. For the UDVA, while the non-inferiority test suggested that the LASIK group was superior, the subsequent age- and sex-matched regression analysis did not find a statistically significant difference between the two groups after adjusting for potential confounders, such as the axial length and ACD. Our data indicate that previous LASIK did not negatively impact the visual outcomes of the EDOF IOLs in terms of predictive refractive error, UNVA, and UDVA when compared with the non-LASIK eyes in this cohort.

IOL power formulas use biometric measurements to predict the refractive outcomes of different IOLs. Measurement methods can be classified as vergence, ray tracing, artificial intelligence, or a combination of these methods [8]. These methods have been employed to predict the postoperative effective lens position and anticipate refractive outcomes, with the aim of minimizing predictive errors [9]. The traditional power calculation model relies on the axial length, corneal K value with only the anterior radius, and ACD for prediction [10]. Current IOL formulas include not only the three variables mentioned above but also lens thickness, central corneal thickness, and white-to-white corneal diameter. For example, the SRK/T formula is one of the most widely used methods for calculating the IOL [8]. In a study by Ota et al., which used the SRK/T formula to calculate the same type of EDOF IOL used in our study, 38–61% of the eyes were within 0.3 D of the prediction error. The discrepancy observed between this study and ours may be attributable to variations in the axial length between Ota et al.’s series (mean: 24.0 ± 1.3 mm, 25.4 ± 1.8 mm) and our dataset (mean: 25.6 ± 1.8 mm) [11]. In an investigation by Jeon et al., which used the SRK/T formula alongside newer formulas such as Barrett Universal II, Kane, and Olsen to calculate the Vivity EDOF IOLs (Alcon, Fort Worth, TX, USA) in patients with a mean axial length of 23.53 mm, a mean absolute prediction error of approximately 0.3 D was reported across all formulas, with the SRK/T formula exhibiting a larger standard deviation [12]. Despite the larger standard deviation, the SRK/T formula remains a reliable choice for calculating the EDOF IOL.

The consideration of not using presbyopia-correcting IOLs in patients with cataract who have a history of LASIK has been discussed in a previous review [13]. This discussion arises from the potential for greater residual refractive errors due to less predictable IOL power calculations compared with non-LASIK eyes. Such unpredictability can lead to unsatisfactory visual outcomes or necessitate re-enhancement procedures [13]. To address inaccuracies in corneal radius and keratometric measurements following refractive surgery procedures, several specialized IOL power calculation formulas have been introduced. Among these, the Haigis-L formula has gained popularity because it does not require previous refractive surgery data [14]. In the study by Abulafia et al., which examined patients with a history of LASIK or photorefractive keratectomy, the mean absolute prediction error was 0.68 ± 0.45 D via the Haigis-L method, 0.63 ± 0.48 D via the Shammas method, and 0.52 ± 0.43 D using the Barrett true-K formula [4]. These values were similar to the 0.65 ± 0.26 D observed in our study, which used the Haigis-L formula.

An interesting observation from our results pertains to the uncorrected distance visual acuity (UDVA) outcomes. Specifically, the LASIK group exhibited a mean UDVA of 0.300 ± 0.362 logMAR, which was notably worse than the 0.117 ± 0.156 logMAR observed in the non-LASIK group. The 95% confidence interval for the difference (0.0033 to 0.3585) encompassed the non-inferiority margin of 0.1 logMAR, indicating that the LASIK group could not be confirmed as non-inferior to the non-LASIK group in this parameter. While the regression analysis did not reveal a statistically significant difference between the groups after adjusting for confounders, this finding raises important considerations. One possible explanation is the presence of subtle corneal irregularities or higher-order aberrations (HOAs) induced by prior LASIK, which may impact the distance vision quality despite acceptable refractive outcomes. LASIK surgery, especially when performed years prior with older technologies, can result in postoperative corneal asphericity and induction of coma aberrations, both of which can degrade the image quality under scotopic conditions and reduce the UDVA even when refraction is close to plano [15,16]. In contrast, non-LASIK eyes typically preserve a more regular corneal shape, which may contribute to sharper unaided distance vision following the EDOF IOL implantation. Additionally, the significantly longer axial lengths and flatter corneal curvatures seen in the LASIK group could have influenced the effective lens position (ELP) or created minor defocus, leading to slight reductions in the UDVA. Previous studies have shown that small prediction errors in ELP estimation can have a disproportionate impact on the UDVA, particularly in eyes with long axial lengths [17]. It was consistent with our study: in the regression analysis, after adjusting for confounders, the LASIK and non-LASIK groups had no difference in terms of UDVA, but the axial length had a significant influence on the UDVA. Our study also revealed that the LASIK group may have a better UNVA and a greater prediction error compared with the non-LASIK group, which could be proof that small prediction errors in the ELP estimation could cause more minor defocus toward the minus degree of refraction, which mimics mild myopic eyes and therefore cause lower UDVA and better UNVA in the LASIK group.

Our study has some limitations. First, our samples were retrospectively selected, presenting a limitation regarding sample size. The relatively small sample size, particularly in the LASIK group, may limit the generalizability of the results. Moreover, after matching the groups based on age and sex, most of the data could not be used. Second, differences in the preoperative demographics and clinical characteristics, including the axial length and ACD, between the groups could introduce potential errors in data interpretation. Third, the follow-up period of one month after surgery may be short for EDOF IOLs because EDOF IOLs may have better visual outcome after a longer follow-up period. Fourth, additional parameters that are important measures of visual quality and satisfaction, such as contrast sensitivity, wavefront aberration, and subjective questionnaires for photic phenomena, such as halo and glare, were excluded. Fifth, the SRK/T formula is a reliable choice for calculating the EDOF IOL; however, it is an early-generation formula. Now, we have more modern formulas which can improve the refraction prediction accuracy, such as the Barrett True-K formula. Due to the retrospective nature of the study, we could not assess the result by utilizing these modern formulas. Further prospective studies with larger cohorts, longer follow-up periods, modern formulas, and comprehensive assessments are warranted to validate our findings.

## 5. Conclusions

In conclusion, our study demonstrated that previous LASIK may have no discernible effect on the visual performance of presbyopia-correcting EDOF IOLs with respect to the absolute refractive error, UNVA, and UDVA. For further validation, longer follow-up and larger-scale or multicenter studies are required to ensure the robustness and generalizability of our results in diverse clinical settings.

## Figures and Tables

**Table 1:** Demographic and clinical characteristics of patients with and without LASIK in the original and propensity-matched cohorts.

Original Data			
Previous LASIK	LASIK (18 Eyes)	Non-LASIK (303 Eyes)	
	Mean ± SD	Mean ± SD	*p* Value
Preop CDVA (logMAR)	0.44 ± 0.27	0.32 ± 0.29	0.0754
Age (years)	50.78 ± 7.14	60.95 ± 8.34	<0.0001 *
Sex (M/F)	7/11	114/189	0.914
Target refraction (D)	−0.59 ± 0.38	−0.57 ± 0.30	0.8403
AXL (mm)	24.73 ± 1.66	27.60 ± 1.39	<0.0001 *
ACD (mm)	3.55 ± 0.35	3.18 ± 0.43	0.0005 *
Sphere (D)	−6.07 ± 5.35	−2.08 ± 4.76	0.0007 *
Cylinder(D)	−1.11 ± 0.8	−1.2 ± 0.90	0.6894
Flat K (D)	37.97 ± 2.15	43.44 ± 1.40	<0.0001 *
Steep K (D)	38.71 ± 2.16	44.33 ± 1.50	<0.0001 *
IOL power (D)	20.08 ± 2.85	18.39 ± 4.80	0.0289
**Matched Data**			
**Previous LASIK**	**LASIK (17 Eyes)**	**Non-LASIK (49 Eyes)**	
	**Mean ± SD**	**Mean ± SD**	***p* Value**
Preop CDVA (logMAR)	0.45 ± 0.27	0.34 ± 0.29	0.1879
Age (years)	51.47 ± 6.71	52.08 ± 6.26	0.7345
Sex (M/F)	7/10	21/28	0.9038
Target refraction (D)	−0.66 ± 0.26	−0.55 ± 0.29	0.2065
AXL (mm)	27.57 ± 1.43	25.63 ± 1.8	0.0001 *
ACD (mm)	3.57 ± 0.36	3.34 ± 0.37	0.0307 *
Sphere (D)	−6.28 ± 5.44	−4.82 ± 4.88	0.3063
Cylinder (D)	−1.12 ± 0.83	−1.38 ± 1.11	0.3715
Flat K (D)	38.07 ± 2.18	43.13 ± 1.55	<0.0001 *
Steep K (D)	38.82 ± 2.17	44.46 ± 1.81	<0.0001 *
IOL power (D)	20.09 ± 2.93	15.83 ± 4.83	<0.0001 *

* *p* value < 0.05; CDVA, corrected distance visual acuity; logMAR, logarithm of the minimum angle of resolution; AXL, axial length; ACD, anterior chamber depth; LASIK, Laser Assisted In Situ Keratomileusis; M, male; F, female; D, diopter; K, keratometry: the measurement of the corneal curvature/power; SD, standard deviation; IOL, intraocular lens.

**Table 2:** Non-inferiority tests of predicted refractive errors and postoperative visual acuity between the LASIK and non-LASIK groups at one month post-operation.

	LASIK (SD) n = 17	Non-LASIK (SD) n = 49	Non-Inferiority Margin	95% CI	Outcome
UNVA (logMAR)	0.315 (0.305)	0.736 (1.307)	0.1	−0.8143, −0.0269	Not inferior
UDVA (logMAR)	0.300 (0.362)	0.117 (0.156)	0.1	0.0033, 0.3585	Non-LASIK is inferior to LASIK
Prediction error (D)	0.656 (0.264)	0.551 (0.287)	0.5	−0.0442, 0.2541	Not inferior

UDVA, uncorrected distance visual acuity; UNVA, uncorrected near visual acuity; logMAR, logarithm of the minimum angle of resolution; CI, confidence interval; LASIK, Laser Assisted In Situ Keratomileusis; D, diopter; SD, standard deviation.

**Table 3:** Age- and sex-matched regression analysis by using the model of generalized estimating equations (GEEs), showing no significant superiority between the LASIK and non-LASIK groups at one month post-operation.

Dependent Variable: PostopUDVA							
Parameter	Level	Est.	SE	95% CI		Z	*p* Value
Intercept		3.4494	2.2501	−0.961	7.860	1.53	0.1253
AXL		−0.1076	0.0508	−0.207	−0.008	−2.12	0.0341
Previous LASIK	1 (yes)	0.1770	0.0952	−0.010	0.364	1.86	0.0631
ACD		0.1443	0.1278	−0.106	0.395	1.13	0.2590
Sphere		0.0140	0.0119	−0.009	0.037	1.17	0.2413
Steep K		−0.0221	0.0290	−0.079	0.035	−0.76	0.4451

UDVA, uncorrected distance visual acuity; AXL, axial length; ACD, anterior chamber depth; CI, confidence interval LASIK, Laser Assisted In Situ Keratomileusis; K, keratometry: the measurement of the corneal curvature/power; Z, z-score; SE, standard error; Est., estimates.

## Data Availability

The original contributions presented in this study are included in the article. Further inquiries can be directed to the corresponding author.

## Abbreviations

The following abbreviations are used in this manuscript:

ACD	Anterior chamber depth
CDVA	Corrected distance visual acuity
D	Diopter
EDOF	Extended depth of focus
IOLs	Intraocular lenses
K	Corneal curvature
LASIK	Laser-assisted in situ keratomileusis
logMAR	Logarithm of the minimum angle of resolution
SE	Spherical equivalent
SRK/T	Sanders Retzlaff Kraff/theoretical
UDVA	Uncorrected distance visual acuity
UNVA	Uncorrected near visual acuity
